# Stress Responses in Dressage Horses: Insights from FEI Noseband Measurements Across National Competition Levels

**DOI:** 10.3390/ani16030518

**Published:** 2026-02-06

**Authors:** Simona Fialová, Dana Kuřitková, Eva Sobotková

**Affiliations:** 1 Victor Kaplan Department of Fluids Engineering, Brno University of Technology, 616 69 Brno, Czech Republic; fialova@fme.vutbr.cz; 2 Department of Animal Breeding, Faculty of Agronomy, Mendel University in Brno, 613 00 Brno, Czech Republic

**Keywords:** dressage, welfare, FEI noseband measuring device (FNMD), equine stress behavior, competition level, performance stress

## Abstract

This pilot study investigated stress-related behaviors in 238 dressage horse–rider combinations competing at national levels from Elementary (A) to Grand Prix (GP). Noseband tightness was assessed on-site using the FEI noseband measuring device (FNMD), and all but two horses complied with FEI regulations. Video-based analysis quantified conflict behaviors including mouth opening, tail swishing, and changes in head–neck position. Because noseband tightness showed minimal variation, no meaningful association with behavioral expression could be determined. In contrast, competition level had a clear influence on both the frequency and type of stress-related behaviors. Horses in lower-level classes displayed a wide range of behaviors at relatively low frequency, while horses in higher-level tests showed fewer behavior types but exhibited them more often, with mouth opening becoming the predominant indicator. Horses ridden in a double bridle generally demonstrated higher proportions of conflict behavior than those ridden in a snaffle. A strong negative correlation between conflict behavior and performance scores was observed only at the lowest levels (A, L). From Medium level upward, judging scores no longer reflected the amount of stress behavior displayed. Overall, the findings indicate that increasing test difficulty is associated with a rise in stress-related behaviors and a narrowing of behavioral expression. These results highlight the importance of considering equipment choice and competition demands when evaluating stress in dressage horses.

## Simple Summary

This study examined visible signs of stress in dressage horses competing at national levels. Using the FEI Noseband Measuring Device, more than 200 horse–rider pairs were evaluated for noseband tightness, type of bridle, and easily observable behaviors such as mouth opening, tail swishing, or changes in head–neck position. Although almost all horses had correctly fitted nosebands, the frequency and type of stress-related behaviors differed across competition levels. Horses in lower-level tests showed a wide range of behaviors, while horses in higher-level classes displayed fewer types of behaviors but showed them more often. As the difficulty of the tests increased, stress indicators also increased. Importantly, at the higher levels, the judges’ scores no longer reflected how much stress behavior the horses showed during their performance.

## 1. Introduction

The most prominent topic in recent years concerning the welfare of horses in competitive sports is so-called stress-related behavior. In general, any sporting use of horses is unnatural and therefore causes stress. Stress can only be eliminated by removing the stressful situation—which is not possible in the case of a sport horse—or through habituation. Habituation is a natural process of reducing the response to repeated stimuli, allowing the organism to conserve energy and focus on new, more important information or changes in the environment. Like any living organism, the horse has a strong ability to adapt to stimuli. Research on pain in animals [1] has shown that animals can use their internal state (e.g., a painful experience) for learning, decision-making, and behavior modification, and they are willing to make sacrifices to avoid these unpleasant states. Equestrian training is based on exploiting this ability. According to behavioral scientists, there are four methods of motivation:

- Negative reinforcement
- Positive reinforcement
- Positive punishment
- Negative punishment

Most equestrian sports training relies mainly on techniques classified as “negative” in learning theory. The most common is negative reinforcement. These methods often produce clear and consistent responses, so they are widely used. The principle is simple: the rider applies pressure, such as with the leg, spur, or bit. The pressure is released when the horse performs the desired response. During early learning, horses may show temporary behaviors linked to discomfort or uncertainty. These include tail swishing, mouth opening, or evasive head–neck positions. Such behaviors are natural reactions to pressure and are relevant indicators of ridden behavior.

This study does not examine training methods. It focuses only on visible behaviors that may be associated with stress during ridden work. Stress in horses can be assessed scientifically in several ways. Animals experiencing stress may display abnormal behaviors [2,3], excessive hormone release [4], decreased performance [5], or various health issues [6]. Physiological measurements, such as cortisol levels in blood [4], saliva [7], or fecal metabolites [8] provide additional information. Heart rate and respiration are also useful indicators [9]. External behavioral signs [1,3] are easy to observe, even for less experienced individuals. For this reason, they are widely discussed in relation to equine welfare.

According to FEI rules, the goal of dressage is a horse that responds to subtle aids without resistance [10]. Horses must perform physically demanding exercises requiring coordination, balance, and strength. They do so in competition environments that may be unfamiliar. Equipment such as the saddle and bridle applies various pressures to the body. Bit pressure on the tongue and lower jaw can cause some horses to open their mouth to relieve the bit pressure. The noseband, a standard part of the bridle, limits excessive mouth opening and stabilizes the bit.

Several studies have examined the distribution of pressure under different parts of the bridle. These studies report the highest pressure behind the ears [11]. Significant pressure also occurs on the lower jaw where the noseband or curb chain lies. Pressure on the nasal bone tends to be lower [11]. Measurements with sensors show that mouth opening changes the pressure applied by the noseband [11]. To support standardized checks, the FEI introduced a certified device for measuring noseband tightness [12]. Its purpose is to ensure that tightness at rest remains within acceptable limits. Since the noseband tightness checks at its competitions were introduced, the focus was on the highest levels of the dressage sport. The main study was presented by Australian researchers about the Amsterdam competition in 2025 [13].

Questions remain about how noseband tightness and bit pressure influence behavioral expression. Some studies report that horses with tighter nosebands open their mouths less frequently. Mouth opening is one of the most visible indicators of discomfort. A reduction in this behavior, however, may not indicate the absence of stress. It may simply reflect a change in how stress is expressed. Rein contact can be described using measurable pressure values. One study reports bit pressures corresponding to forces from approximately 0.2 to 8 kg, depending on bit type and rein tension [14].

This study does not aim to evaluate welfare comprehensively. It documents visible behavioral indicators across different competition levels. It also highlights areas where further research is needed. A similar study on a smaller number of horses was conducted at British local competitions by the authors [15]. Their findings concluded that conflict behaviors in horses were nearly universal (precisely 94.8%) regarding mouth opening across all levels and bridle types—a truly dramatic result.

The aim of this study was to characterize stress-related behaviors in dressage horses at different national competition levels (Table 1). It also aimed to assess their association with noseband type, bridle configuration, and test performance.

## 2. Materials and Methods

**Figure 1:**
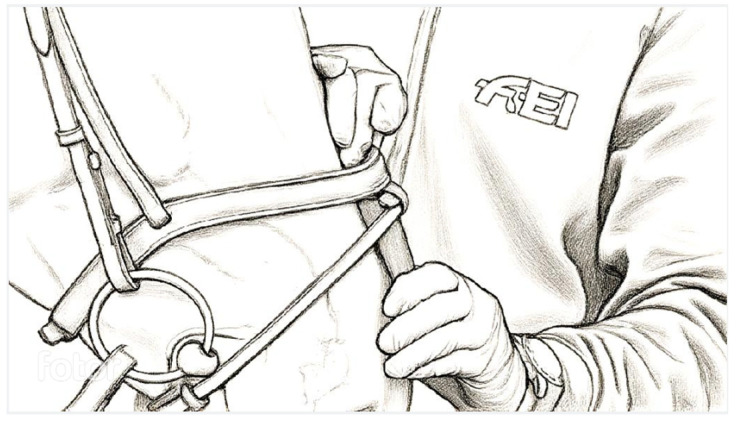
Use of FEI noseband measuring device (FNMD)—English crank noseband.

**Figure 2:**
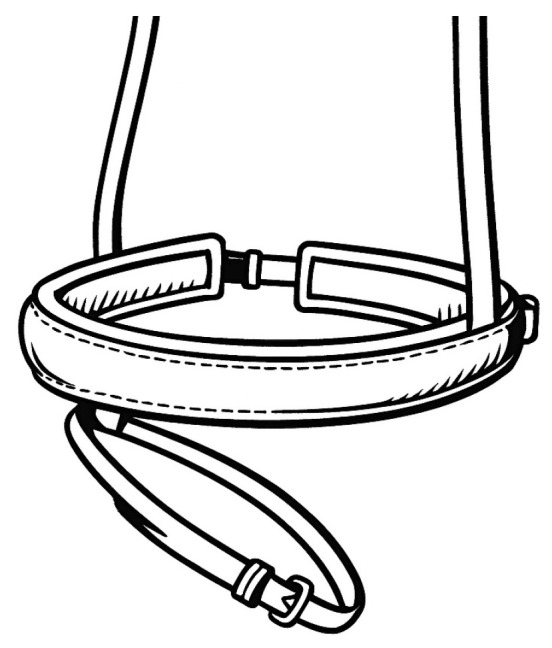
Swedish crank noseband with leverage buckle system.

**Figure 3:**
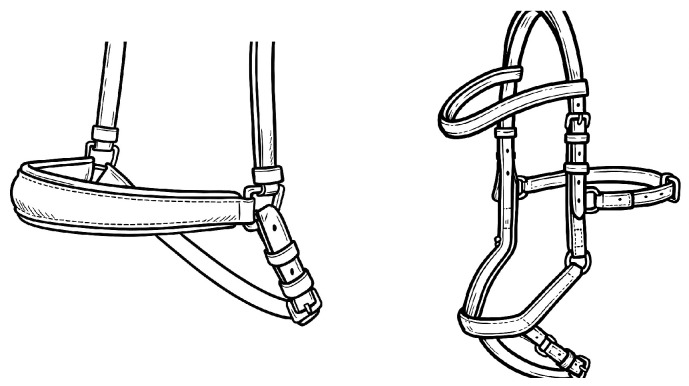
Drop/Micklem noseband.

The measurements [16] were carried out over several competition days. Noseband tightness was evaluated on-site in a yes/no format (whether the measuring device passed or not), along with the type of noseband (Figure 1, Figure 2 and Figure 3) and bit.

### 2.1. Noseband Tightness Measurements

All horses were checked during warm-up and preparation for the competition (see Figure 1). The measurement results were clearly presented in graphs and tables, evaluating all correlations with respect to the level of difficulty and the horses’ negative responses to rider aids.

### 2.2. Video Analyses

**Figure 4:**
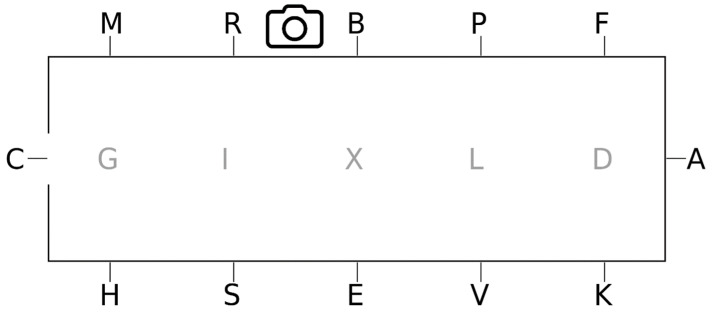
Dressage arena with the marked-up position of the camera.

Video documentation was recorded during the competition from a single point on the long side of the arena (between R and B) with sufficient resolution, and stress-related behaviors were subsequently analyzed over time (see Figure 4).

To avoid starting the evaluation with a negative note, it should be emphasized that out of all 238 observed pairs (Table 2), only 2 were flagged for having a noseband tightened beyond the recommended level, requiring greater effort to pass the FNMD. Both pairs immediately adjusted the tightness on their own initiative, so it can be stated that all assessed pairs had nosebands tightened in accordance with FEI regulations. The number of horses at each level is shown in Table 2.

The relatively small number of horse–rider combinations at the highest competition levels (S*, S**, GP) reflects the nature of national-level competitions, where participation in elite classes is generally limited. Unlike international FEI events, national competitions are characterized by a broad base of entries at lower and medium levels and a substantially smaller number of combinations competing at the highest levels.

### 2.3. Statistics

All analyses were conducted in STATISTICA CZ version 14. Within each competition level, the effect of noseband type on time spent in conflict behavior (stress time) was tested using one-way ANOVA when assumptions were met. When ANOVA was significant (A and L level), Tukey HSD was used. The effect of noseband type on percentage score was analyzed in the same way. Correlations between percentage score and time in conflict behavior were quantified using Pearson’s r; Spearman coefficients are provided where applicable. Statistical significance was set at p ≤ 0.05.

### 2.4. Ethical Considerations and Data Handling

This study consisted solely of non-invasive behavioral observations conducted during regular national dressage competitions, which do not require formal ethical approval under national guidelines. All riders or horse owners provided verbal informed consent prior to participation. Data were recorded in anonymized form, each horse–rider pair was assigned a numerical code, and no identifying information was included in the analysis. The identification key was accessible only to the authors, and all data were stored securely on password-protected institutional servers.

## 3. Results

Results are presented by competition level with descriptive summaries of stress-related behaviors. Because noseband tightness showed insufficient variability (all combinations complied with FEI regulations), meaningful assessment of its tightness association with behavior was not possible; however, there was a meaningful influence on the amount of conflict behavior during the test, see Table 3.

However, the level of competition had a significant impact on the type and frequency of conflict behaviors. The main impact on the percentage scores has conflict behavior in low-level competitions (A and L levels). From M level higher, the percentage scores did not reflect the increasing amount of conflict behavior in the test, since more focus is on the technical presentation of the prescribed movements and figures. This trend in the lack of correlation between higher scores and horses being ridden behind the vertical at national levels [17] and international FEI levels [18] has also been confirmed by other studies focused solely on this phenomenon.

**Figure 5:**
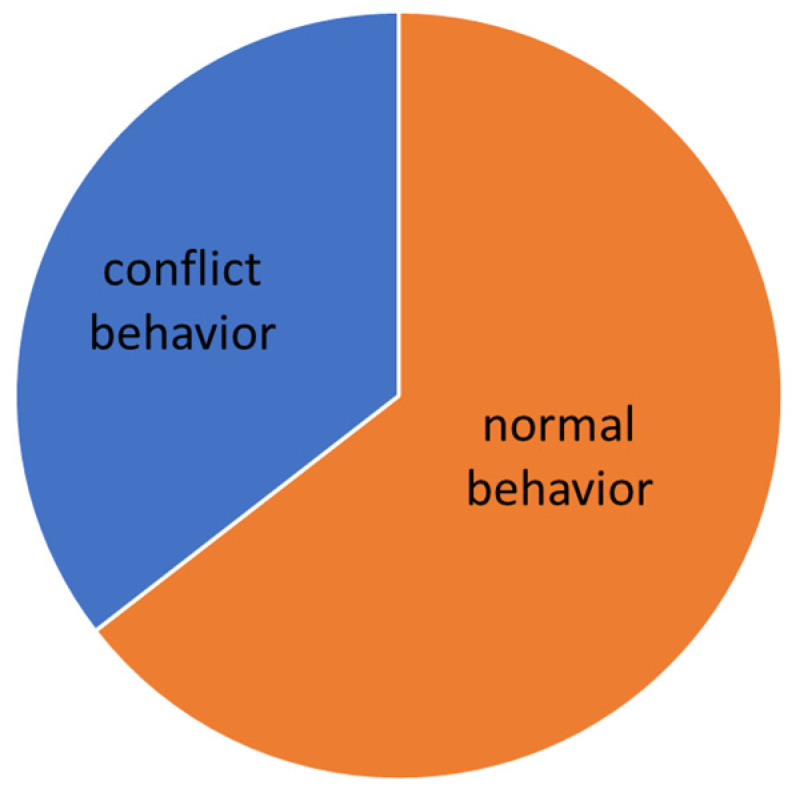
Total time of conflict behavior in the Elementary level (A).

**Figure 6:**
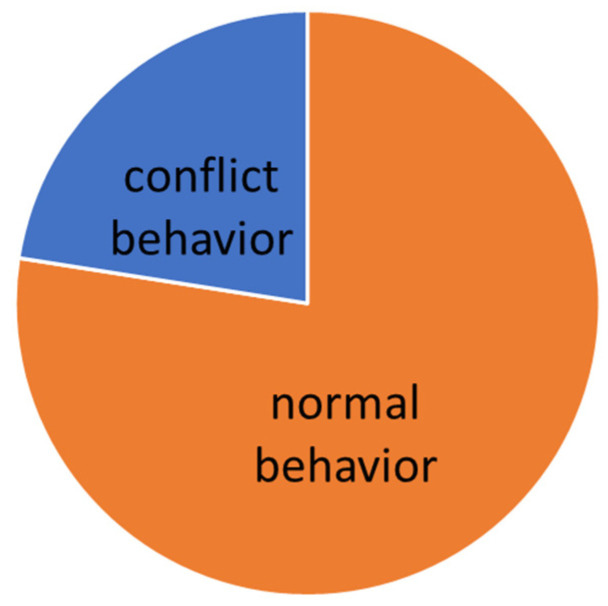
Total time of conflict behavior in the Basic level (L).

**Figure 7:**
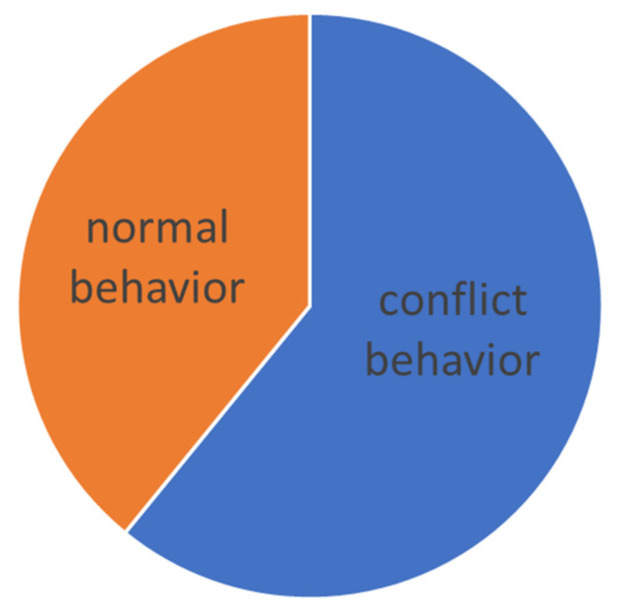
Total time of conflict behavior in the Medium level (M).

**Figure 8:**
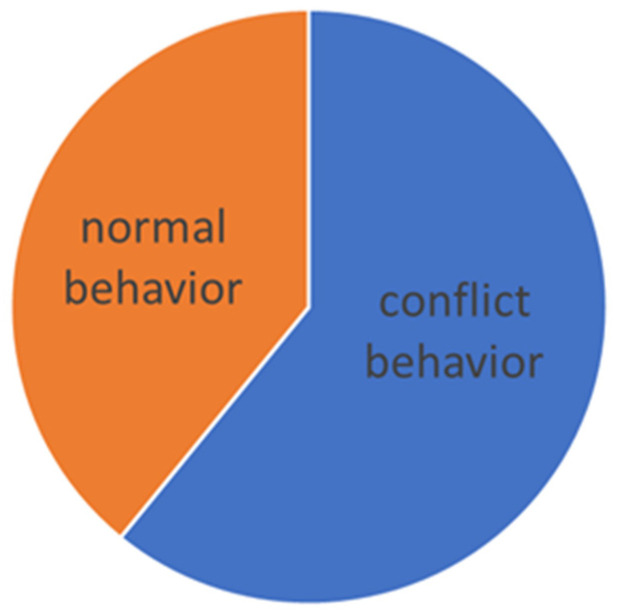
Total time of conflict behavior in the Intermedium level (S* + S**).

**Figure 9:**
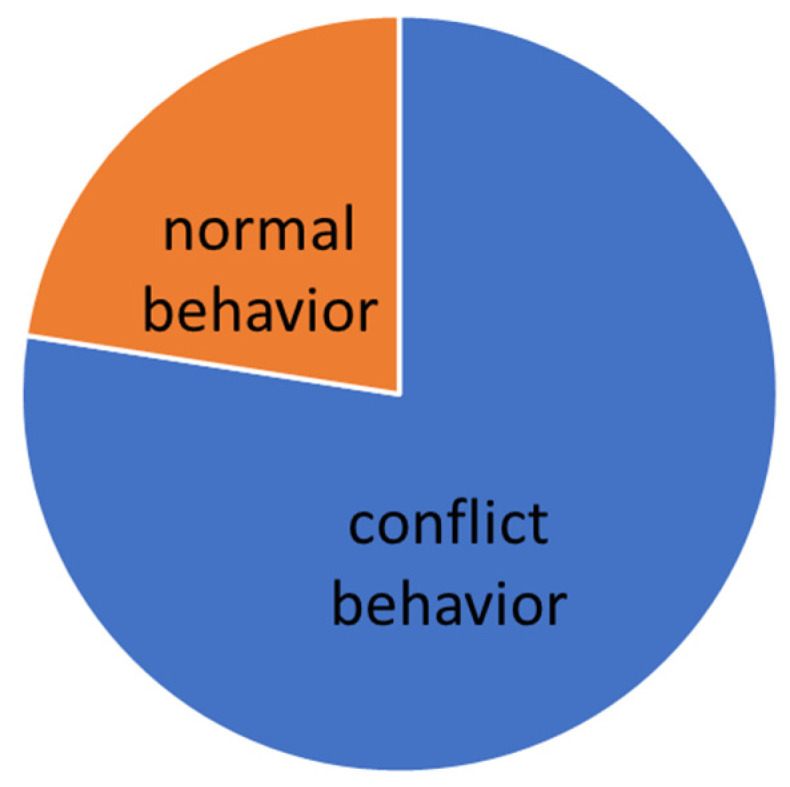
Total time of conflict behavior in the Grand Prix level.

The evaluation was carried out according to the individual levels of difficulty. The manifestation of conflict behavior distribution to the normal behavior in different levels of dressage classes is presented graphically (see Figure 5, Figure 6, Figure 7, Figure 8 and Figure 9).

**Figure 10:**
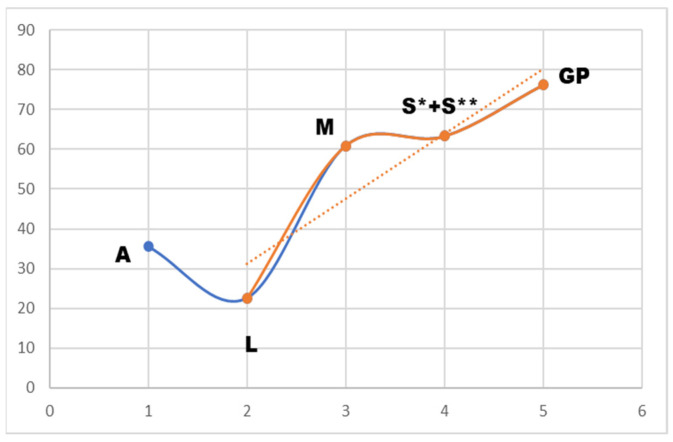
Growing tendency of conflict behavior in the classes according to difficulty. Blue line shows the drop in stress behavior due to low difficulty and more gained experiences of the horses (first and second year of competing), while orange shows increasing stress behavior corresponding to the increasing difficulty.

Figure 10 illustrates a clear increasing trend in the amount of conflict behavior with rising test difficulty. However, this pattern was not reflected in the performance scores, as shown in Table 4.

### 3.1. Summary of Statistical Outcomes (Per Level)

- **Elementary (A)**

Noseband effect on stress time is significant: $F\left(2.105\right)=7.354,p=0.00103$.

Post hoc: English>Swedish; Micklem intermediate.

KW significant: $\chi^{2}=8.121,p=0.017$.

Correlation score × time: $r=-0.445,p=1.43\times 10^{-6}$.

- **Basic (L)**

Noseband effect is significant: F(2.48)=5.265,p=0.00856.

Post hoc: English>Swedish; $Micklem\;NS\left(N=4\right).$

KW significant: $\chi^{2}=8.159,p=0.0169$.

Correlation: $r=-0.454,p=9.17\times 10^{-4}$.

- **Medium (M)**

Noseband effect not significant: $F\left(2.37\right)=0.302,\;p=0.741$.

Correlation: r = −0.126, p = 0.422.

- S* + S**

Noseband effect not significant for stress time: F(2.17)=0.063,p=0.939;

KW:p=0.985.

Effect on score is significant: $F\left(2.17\right)=5.547,p=0.014,$ Swedish>English.

Correlation:r=−0.127,p=0.593.

- **Grand Prix (GP)**

Not enough cases for noseband comparison.

Correlation: r = −0.285, p = 0.584.

- **Across all levels**

Level effect is significant for stress time: $F\left(4.225\right)=7.306,\;p=1.51\times 10^{-5}$.

Tukey: Medium > A, Medium > L.

Effect on percentage score borderline: F(4.222)=2.394, p=0.0515.

No corresponding relationship between conflict behavior and awarded marks was observed in the higher-level classes, see Table 4.

### 3.2. A Level

On A level (elementary movements in walk, trot, and canter), where the use of a double bridle is forbidden, the general number of total conflict behavior sets to 35.5%. The variety of conflict behavior seems to be typical for “green” horses, see Table 5. Interesting findings come from the statistics, where there is a good correlation between the lower scores and time spent in conflict behavior during the test (Table 4). It was also proven that the effect of the chosen noseband type, where the most restrictive type of noseband (Swedish crank with flesh—Figure 3) caused lower signs of stress in the performance (Table 3).

### 3.3. L Level

On L level (lateral work in trot, simple changes, counter canter), where the use of a double bridle is forbidden, the general number of total conflict behavior decreases to 22%. The variety of conflict behavior remained typical for “snaffled” horses, see Table 6. Also, on the L level, there is a good correlation between the lower scores and time spent in conflict behavior during the test. Proved was also the effect of the chosen noseband type, where the most restrictive type of noseband (Swedish crank with flesh—Figure 3) caused lower signs of stress in the performance.

### 3.4. M Level

**Figure 11:**
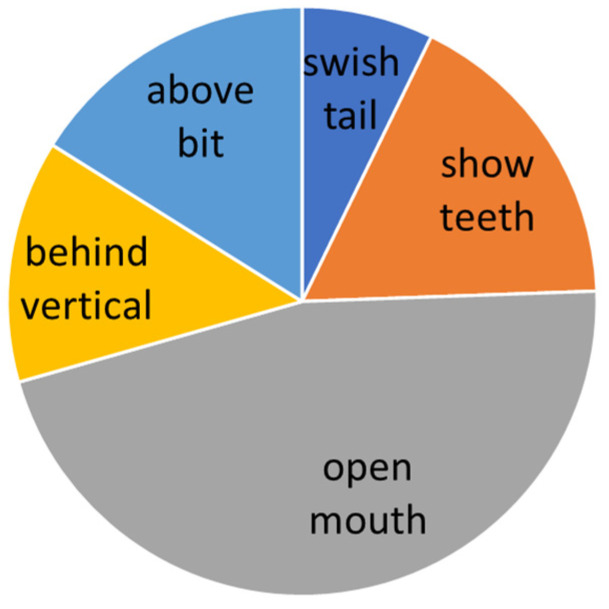
Conflict behavior performed on the snaffle in M level.

**Figure 12:**
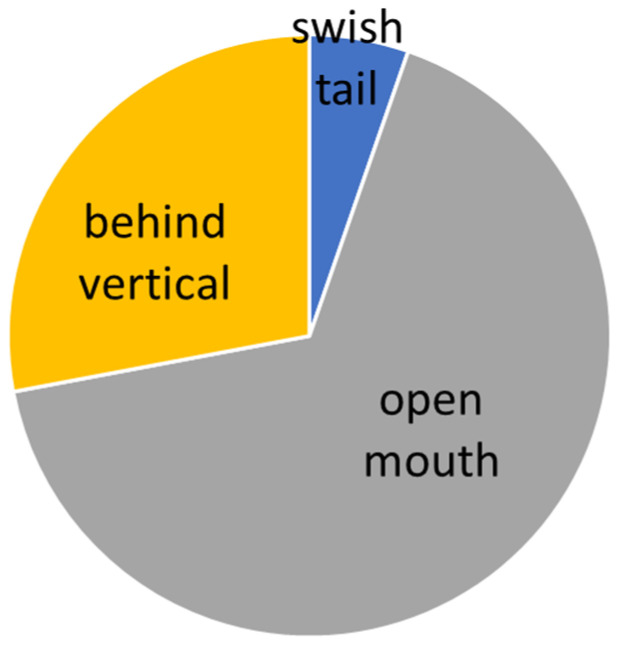
Conflict behavior performed on the double bridle in M level.

On M level (lateral work in trot and canter, counter canter, flying changes), where the use of a double bridle is optional, the general number of total conflict behavior jumps up to 61%, while horses on the double bridle showed 68% when horses on snaffle bit only 57%, see Table 7. What has changed is the layout of conflict behavior when the open mouth and tail swishing were abandoned for “doublebridled” horses, while “lower-levels” signs of stress remained for “snaffled” horses, see comparison in Figure 11 and Figure 12. Statistics showed from M level higher, low to no correlation of the noseband choice with the results (Table 3). The same tendency was visible in between the resulting scores and the amount of conflict behavior during the test (Table 4).

### 3.5. S* + S** Level

**Figure 13:**
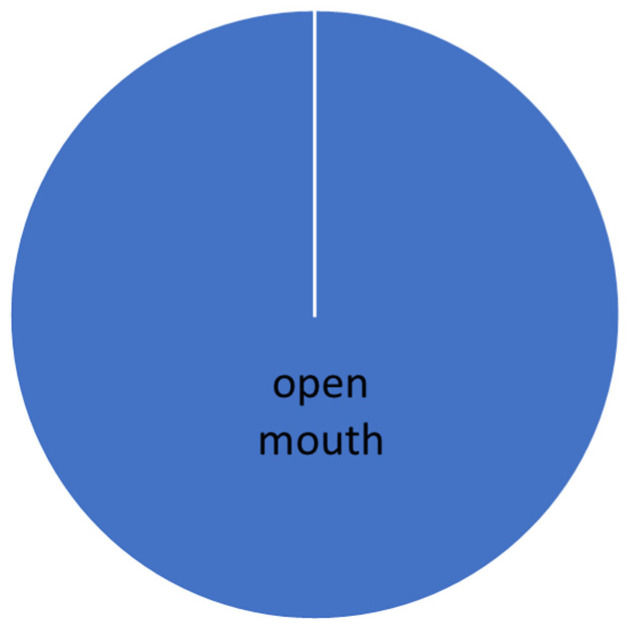
Conflict behavior on the snaffle in S* and S** level.

**Figure 14:**
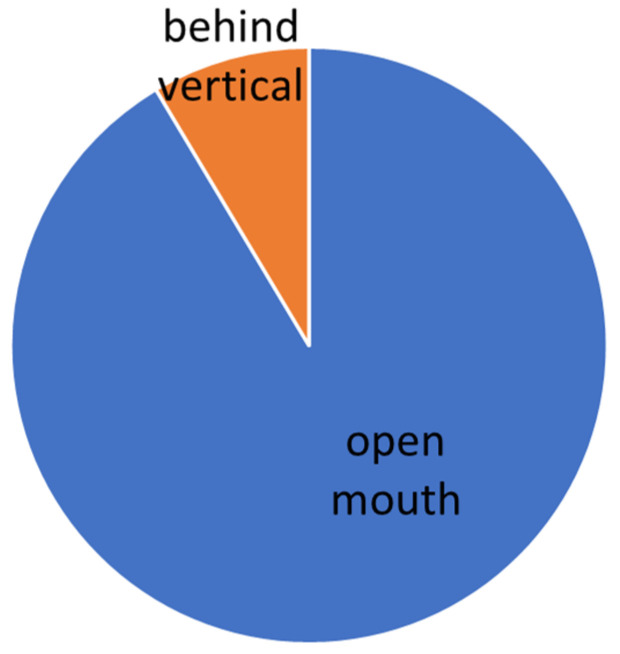
Conflict behavior on the double bridle snaffle in S* and S** level.

On S level (lateral work in trot and canter, series of flying changes (4, 3, 2), canter pirouettes), where the use of double bridle is optional, the general number of total conflict behavior increases to 63.3%. The layout of conflict behavior has significantly changed and reduced to open mouth and behind vertical, as shows Table 8. What is interesting is the absence of horses behind vertical for “doublebridled” horses, so their stress was shown only by open mouth (see comparison in Figure 13 and Figure 14), where only differentiation of conflict behavior is shown. No link between the percentage results and time spent in conflict behavior was confirmed (see Table 4).

### 3.6. GP Level

**Figure 15:**
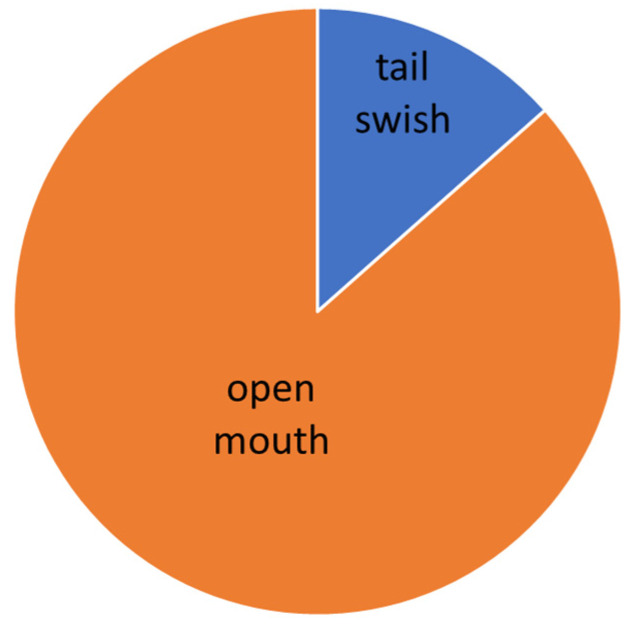
Conflict behavior on the snaffle in GP level.

**Figure 16:**
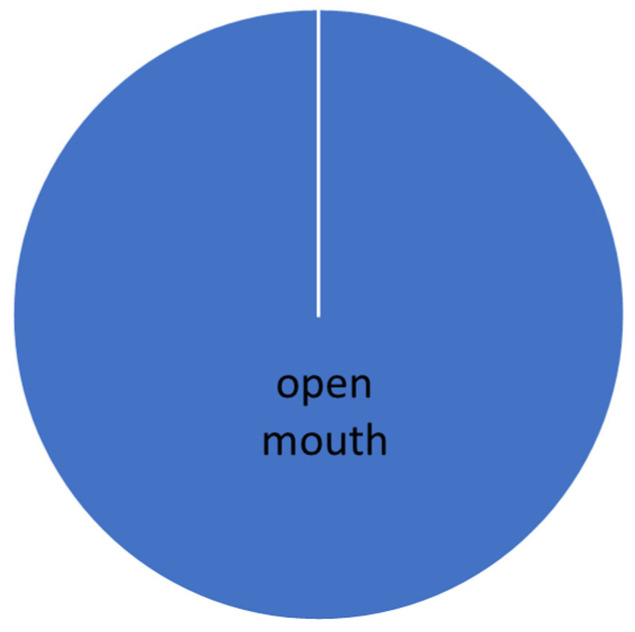
Conflict behavior on the double bridle in GP level.

On GP level (lateral work in trot and canter, series of flying changes (2, 1), canter pirouettes, piaff, passage), where the use of double bridle is optional, the general number of total conflict behavior increases to 76.2%. The layout of conflict behavior has significantly changed and reduced, visible in Table 9. What is interesting is the absence of horses’ tail swishing for “doublebridled” horses, so their stress was shown only by open mouth, see comparison in Figure 15 and Figure 16. No correlation between the percentage results and time spent with conflict behavior was confirmed, see Table 4.

## 4. Discussion

Stress in competition horses is influenced by performance, environmental change, and management conditions. These factors may contribute to behavioral variation and limit attribution of stress indicators solely to the ridden test.

The results show a clear increase in stress-related behaviors with rising test difficulty. A decrease was observed only between the Elementary and Basic levels, likely reflecting early habituation and improved understanding of basic aids. At the lowest levels, horses showed a broad range of behaviors, including head–neck issues, vocalization, reluctance to move, and contact-related responses.

From Medium level onward, conflict behaviors increased markedly. Higher levels were characterized by fewer behavior types but higher frequency. Mouth-related behaviors dominated. Behaviors linked to misunderstanding or separation largely disappeared, suggesting a shift from general stress responses to contact-related indicators under higher technical demands.

Noseband tightness could not be evaluated due to minimal variation, but noseband type influenced behavior in lower levels. At levels where double bridles were optional, horses ridden in a double bridle showed more conflict behavior. Performance scores did not reflect the amount of stress behavior shown from the Medium level upward.

These findings demonstrate that test difficulty and equipment choice influence the expression of stress-related behaviors in dressage horses. Further controlled studies are needed to isolate equipment effects, training factors, and management conditions and to support welfare-oriented recommendations for all competition levels.

## 5. Conclusions

This study demonstrates that stress-related behaviors in dressage horses increase with test difficulty. At lower levels, horses show a wide range of behaviors, while at higher levels, the behavioral spectrum narrows and mouth-related responses become dominant. Noseband tightness could not be assessed due to minimal variation, but noseband type affected behavior in lower levels. Where double bridles were optional, horses ridden in a double bridle showed more conflict behavior. From Medium level upward, performance scores did not reflect the amount of stress-behavior shown. These results indicate that test difficulty and equipment choice influence the expression of stress in competition horses and should be considered when evaluating welfare. Further controlled research is needed to clarify how equipment, training, and management conditions shape behavioral stress responses.

## Figures and Tables

**Table 1:** Description of requirements for each test level, together with the prescribed type of bridle.

Elementary (A)	basic gaits without collection or extension, simple lines	snaffle
Basic (L)	A + collected and extended gaits, counter canter, simple change in canter, leg-yield and half-pass in trot, turn on the haunches in walk	snaffle
Medium (M)	L + half-pass in canter, flying change, half-pirouette in walk	snaffle/curb
Intermedium (S*)	M + half-pirouette in canter, series of flying changes every 4 and 3 strides	snaffle/curb
Advanced (S**)	S* + full pirouette in canter, series of flying changes every 2 strides	snaffle/curb
Grand prix (GP)	S** + Series of flying changes every stride, piaffe, passage, pi-pa transitions	snaffle/curb

**Table 2:** Number of competing horse–rider combinations in each level.

Elementary (A)	110 horses	snaffle
Basic (L)	51 horses	snaffle
Medium (M)	50 horses	snaffle or curb
Intermedium (S*)	12 horses	snaffle or curb
Advanced (S**)	9 horses	snaffle or curb
Grand prix (GP)	6 horses	snaffle or curb

**Table 3:** Noseband style effect on the conflict behavior time for each level (indexes a, b show the differentiation between the values. SE—standard error of the Mean value; N—number of cases).

Parameter	Mean Value A ± SE (s)	Mean Value L ± SE (s)	Mean Value M ± SE (s)	Mean Value S ± SE (s)	Mean Value GP ± SE (s)
Noseband					
English (Figure 1)	117.8 ± 13.8 ^b^ N-38	114.7 ± 22 ^b^ N-18	156 ± 35.8 ^a^ N-17	221.8 ± 42,2 ^a^ N-13	
Swedish (Figure 2)	48.7 ± 11.7 ^a^ N-53	26.3 ± 17.3 ^a^ N-29	181.7 ± 33 ^a^ N-20	195.4 ± 62.1 ^a^ N-6	248.9 ± 43.7 ^a^ N-6
Drop/Micklem (Figure 3)	81.2 ± 20.6 ^ab^ N-17	95.8 ± 46.6 ^ab^ N-4	220.6 ± 85.3 ^a^ N-3	222.3 ± 152 ^a^ N-1	

**Table 4:** Correlation between the conflict behavior and result (r—correlation coefficient; *p*-value).

Competition Level	Mean Value of All Results (%)	Mean Value of Conflict Behavior (s)	Nr. of Horses	r (%, s)	*p*
A	61.54	78.12	108	−0.445	<0.001
L	61.53	58.63	50	−0.454	<0.001
M	63.35	196.52	43	−0.126	0.422
S* + S**	64.01	213.93	20	−0.127	0.593
GP	61.13	248.92	6	−0.285	0.584

**Table 5:** Relative time spent in conflict behavior during A level dressage test.

	Snaffle
**Conflict behavior**	**35.5%**
Open mouth	31.1%
Above bit (not through the poll)	44.2%
Short in neck	2.5%
Tail swishing	0.5%
Behind vertical	20%
Resistance	2.5%
Whining	1.4%

**Table 6:** Relative time spent in conflict behaviors during L level dressage test.

	Snaffle
**Conflict behavior**	**22.5%**
Open mouth	44.2%
Above bit (not through the poll)	27%
Short in neck	7.8%
Tail swishing	4.3%
Behind vertical	15.6%
Resistance	0.6%
Whining	0.6%

**Table 7:** Relative time spent in different type of conflict behavior during M level dressage test.

	General	Snaffle	Double Bridle
**Conflict behavior**	**60.9%**	**57.3%**	**68.1%**
Open mouth	50.4%	26.4%	45.5%
Above bit (not through the poll)	12.3%	9.2%	0%
Busy mouth (play with lips)	13.1%	9.9%	0%
Tail swishing	6.7%	4.1%	3.6%
Behind vertical	16.5%	7.6%	19.1%

**Table 8:** Relative time spent in different types of conflict behavior during S level dressage test.

	General	Snaffle	Double Bridle
**Conflict behavior**	**63.3%**	**49.7%**	**69.2%**
Open mouth	63.6%	28.2%	69.2%

**Table 9:** Relative time spent in different types of conflict behavior during GP level dressage test.

	General	Snaffle	Double Bridle
**Conflict behavior**	**76.2%**	**33.4%**	**59.4%**
Open mouth	73.9%	85.5%	68.1%
Tail swishing	2.3%	13.6%	0%

## Data Availability

Data is unavailable publicly due to the privacy of participating riders.
